# The German System

**Published:** 1912-11-16

**Authors:** 


### THE GERMAN SYSTEM.

### V.—The Financial Aspect of the German Hospitals.

IN previous articles of this series (THE HOSPITAL, June 24, July 1 and 8, 1911, and February 10, 1912) we gave certain general figures regarding the finances of the German hospitals, and pointed out what were the main features of the system under which the hospitals in the German Empire were maintained. We are now in a position to make further generalisations, and to draw broad conclusions from the mass of facts which are available. In the first place it is desirable to repeat, briefly, the main features which characterise the German system. Hospitals in Germany are in the vast majority of cases communal institutions; a few are purely State-owned and State-supported houses, and a still smaller percentage are private hospitals supported by free grants (excluding, of course, the large number of nursing homes, which, as we have already stated, are purely business undertakings, which cannot for a moment be classed in the same category as ordinary hospitals, even though they are lumped together under the heading of "private institutions" by the German statisticians). There is no general law which applies to all the hospitals, and the administration and regulation of these institutions depend wholly on the correct interpretation of numerous local by-laws and general "customary" principles, which vary in each province.

### THE LATEST STATISTICAL REVIEW.

In Germany the main burden of hospital upkeep, then, falls on the municipalities, and this burden is increasing year by year. Ober-Inspektor Einert, in his interesting paper "Die allgemeinen Krankenhäuser und ihr Betrieb in financieller Beleuchtung," which was read at the last conference of the hospital administrators held at Dresden this year, has broadly outlined the reasons for this progressive increase

### TWO HOSPITAL BUDGETS.

Herr Einert takes as his typical example the case of the Dresden hospitals. In 1849 the municipality took over the city hospital Dresden-Friedrichstadt, and in 1901 the Dresden-Johannstadt hospital. The first has 1,272 beds, and covers an area of 101,000 square metres; the second and smaller hospital occupies 63,450 square metres of ground, and provides 580 beds. The first cost 3,663 marks, the second 9,509 marks per bed so far as costs of building only are concerned. When the expenses of equipment are taken in the cost per bed for the Johannstadt hospital is raised to 10,200 marks.

It is unnecessary to point out the differences in detail, but hospital workers will note with interest that the decreases have been in categories which show a corresponding decrease in English hospital statistics—alcohol for instance —while there has been a general and marked increase all along the line. These figures show very clearly the progressive increase which has taken place, and are interesting when compared with the statistics of a large English institution such as, for instance, the London Hospital or the Bristol Infirmary.

The following comparative statistical table of the finances of the two institutions are taken from Herr Einert's exhaustive tables :

The figures represent :

| | 1884 | 1909 | |
|---|---|---|---|
| | Friedrich-stadt Marks | Friedrich-stadt Marks | Johann-stadt Marks |
| Revenue from patients' payments ... ... | 257,652 | 729,313 | 344,788 |
| Expenditure : | | | |
| Salaries and wages : | | | |
| Medical staff ... ... | 16,550 | 70,724 | 34,276 |
| Dispensing staff ... | — | 14,283 | 8,258 |
| Chaplain and choir ... | 2,803 | 10,716 | 5,687 |
| Administrator ... ... | 16,852 | 41,961 | 33,784 |
| Servants ... ... | 12,208 | 37,977 | 24,649 |
| Laundrymaids ... ... | 4,009 | 14,847 | 9,857 |
| Kitchen ... ... ... | 3,406 | 14,117 | 14,554 |
| Barber ... ... ... | 340 | 750 | 450 |
| Nursing staff ... ... | 21,246 | 97,230 | 50,223 |
| | 77,414 | 302,605 | 181,738 |
| Expenses of Administration : | | | |
| Food and provisions ... | 122,975 | 323,197 | 170,754 |
| Laundry : uniforms ... | 10,098 | 23,844 | 12,801 |
| Soap ... ... ... | 2,505 | 5,231 | 3,523 |
| Domestic necessaries ... | 936 | 2,765 | 1,390 |
| Fuel ... ... ... | 23,839 | 72,410 | 43,175 |
| Lighting ... ... | 9,980 | 31,031 | 19,638 |
| Medicines : drugs ... | 17,539 | 22,596 | 8,497 |
| Alcohol and minerals ... | 7,415 | 5,935 | 1,619 |
| Bandages ... ... | 9,498 | 38,024 | 12,188 |
| Special classes for nurses | — | 862 | 427 |
| Judicial and notarial expenses ... ... | 511 | 56 | 20 |
| Stationery ... ... | 1,517 | 2,220 | 1,458 |
| Library ... ... ... | 300 | 800 | 461 |
| Domestic utensils ... | 7,647 | 9,725 | 5,926 |
| Surgical appliances ... | 1,601 | 15,091 | 4,744 |
| Special departments ... | — | 4,852 | — |
| X-ray departments ... | — | 3,876 | 1,640 |
| Laboratory ... ... | — | 3,342 | 1,331 |
| New laboratories ... | — | 3,360 | — |
| Special library ... ... | — | 1,520 | 997 |
| Dispensary laboratory | — | 120 | 83 |
| Grant to pathological department ... ... | — | 2,782 | 2,495 |
| Bacteriological institute | — | 3,208 | — |
| Upkeep of buildings ... | 19,157 | 48,437 | 22,923 |
| Upkeep of special block ... ... ... | 9,155 | 23,615 | 5,950 |
| Garden ... ... ... | 2,820 | 5,115 | 3,140 |
| Water ... ... ... | 9,572 | 14,586 | 7,565 |
| Insurance fees (under Invalidity Insurance Acts) ... ... ... | — | 4,033 | 2,878 |
| Rates and taxes ... | 1,215 | 1,982 | 1,492 |
| Various (unclassified) ... | 1,575 | 3,998 | 2,650 |
| Personal expenses (unclassified) ... | 77,414 | 303,605 | 181,739 |
| Total expenditure ... | 337,260 | 980,918 | 521,503 |

The Summary.

Abstracting the full tables given, we obtain the following general condensation :

| | 1884 | 1909 | |
|---|---|---|---|
| | Friedrich-stadt Marks | Friedrich-stadt Marks | Johann-stadt Marks |
| Revenue from patients' payments ... ... | 257,652 | 729,313 | 344,788 |
| Expenditure ... ... | 337,260 | 980,918 | 521,504 |
| Grant from municipality | 79,608 | 251,605 | 176,716 |
| Total number of nursing days ... ... | 172,628 | 293,495 | 128,706 |
| Total number of in-patients healed ... | 6,094 | 9,387 | 4,107 |

| | 1884 | 1909 |
|---|---|---|
| Total income (for Income Tax purposes) of population of Dresden ... ... ... | (238,640 pop.) 148,702,451 | (546,400 pop.) 457,132,132 |

### THE FINANCIAL SYSTEM.

Dealing with the question of revenue, Herr Einert points out that under the present system the income of the large institutions is provided for mainly from two sources—patients' payments and the sale of superfluous materials, endowments and other minor extras, and, secondly, municipal grants. The relation between income and expenditure depends mainly on the first source, and it is therefore in the interest of the institution to take care that it obtains the maximum amount from its patients.

To English administrators accustomed to the voluntary principle this may seem a radical revolution of the essential principles of hospital ethics. As a matter of fact, it is nothing of the kind. In preceding articles we have already pointed out that although no patient is admitted free into a municipal hospital in Germany, the percentage of poor patients—that is to say, patients who cannot afford to pay even the relatively low cost of treatment out of their private means—is as high in these institutions as in the average English voluntary hospital.

### A Question of Comparison.

Let us once and for all be clear on this point. The average cost per bed per in-patient per day at St. Thomas's Hospital is 5s. 8d.; the average cost per patient at a large German hospital may be put down at 3s. 6d. Bearing in mind the fact that in the English institution there is no differentiation of class, whereas in the German there is a distinct differentiation whereby the better class patient is charged from 5s. to 8s. per day, and the poorer class from 3s. to 3s. 6d., it is evident that the latter hospital possesses, at least theoretically, a better means of controlling hospital abuse, so far as in-patients are concerned, than the former. Everyone who has had experience of hospital work in this country must admit that under our system many patients come into the wards, especially into the surgical wards, who are able to pay small fees. Their admission is theoretically again an abuse of the hospital privileges which is impossible under the German system. It may be argued that while this is the case, the pay system cuts both ways, and that in German hospitals the really necessitous patient who cannot afford to pay the small fees demanded is robbed of the chance of treatment. That, however, is provided for under the Unterstützungswohnsitzgesetz, the principle of which we have already outlined (The Hospital, July 8, 1911). These patients are paid for by the municipality, and the income from such payments is put down under patients' payments, and is not reckoned as part of the municipal grant. A German hospital superintendent is always keen to pass through his wards the largest number of patients in the minimum time, and although the day room is still a recognised part of every German ward and convalescent homes are still wanting in many hospital establishments, the percentage of patients treated is rapidly rising at each institution. The average length of stay in the wards is perhaps longer than it ought to be, but this figure is largely discounted when we consider the fact that temporary beds are rarely used and that slight cases are treated in the casualty department of the polyclinics and not admitted to the wards. At any rate, those who know German surgery will admit that the best use is made of the time, and that a long average stay is no disadvantage to the patients.

### The Rising Cost of Hospitals—How it Affects the Rates.

Naturally the increasing cost of hospitals owned by municipalities has led to increased grants from these bodies, with the inevitable result of increased rates. The authorities have repeatedly attempted to diminish the cost of upkeep without impairing the efficiency of their institutions—so far without conspicuous success. It has been argued that instead of providing large general hospitals, a series of small hospitals should be established; but the argument that a small hospital costs less in upkeep is an entirely fallacious one when the initial proviso, that the institution must be fully up to date and modern in the best sense of the term, is kept in mind. The eventual solution of the difficulty will probably lie in the establishment of hospital suburbs or cities, as suggested by Sir Henry Burdett. Certainly the building of provisional hospitals and sanatoriums has not materially lightened the work of hospitals and the proportionate expenditure entailed by their upkeep.

### The Suggested Way Out.

Unless some means is found to provide the hospitals with funds the inevitable result will be a raising of the fees charged to patients, with the corresponding result that the revenue derived from patients' payments will decrease. This may seem paradoxical, but it has been proved that in several hospitals where it has been found necessary to raise the charges the admissions of second and first class patients have dropped, owing to the competition with private clinics.

### Conclusions.

It will be seen, from this exposition, that the financial condition of the German hospitals is not so favourable as some of us imagine, notwithstanding the fact that they are rate-supported. The subject of upkeep cost is at present interesting the large section of hospital opinion in the Empire, but it is evident that the public, as a whole, has not yet realised how important it is that the general hospitals should be placed in a position to carry on their work thoroughly and efficiently and unhampered by want of funds. As we have shown, the introduction of social legislation of the type of the Insurance Acts has popularised hospitals in the Empire, but it has, apparently, failed to impress on the public the sense of its obligations to these institutions. It is easy enough to add to the rates, but in course of time increased rates will produce irritation and discontent, the effects of which will very likely produce a reaction unfavourable to the rate-supported hospitals. This danger menaces German hospitals, and it is to be hoped that German hospital workers will find some means of circumventing it without impairing the acknowledged efficiency and usefulness of the splendid institutions they possess.

